# High Frequency of *ERBB2* Activating Mutations in Invasive Lobular Breast Carcinoma with Pleomorphic Features

**DOI:** 10.3390/cancers11010074

**Published:** 2019-01-11

**Authors:** Juan Manuel Rosa-Rosa, Tamara Caniego-Casas, Susanna Leskela, Eva Cristobal, Silvia González-Martínez, Esther Moreno-Moreno, Elena López-Miranda, Esther Holgado, Belén Pérez-Mies, Pilar Garrido, José Palacios

**Affiliations:** 1 CIBER-ONC, Instituto de Salud Carlos III, 28029 Madrid, Spain; susanna.leskela@gmail.com (S.L.); pilargarridol@gmail.com (P.G.); 2 Instituto Ramón y Cajal de Investigación Sanitaria, 28034 Madrid, Spain; tamara880723@hotmail.com (T.C.-C.); evamaria.cristobal@salud.madrid.org (E.C.); bperezmies@gmail.com (B.P.-M.); 3 Department of Medical Oncology, Hospital Ramón y Cajal, 28034 Madrid, Spain; silviagonzalezmartinezbio@gmail.com (S.G.-M.); elemiranda@hotmail.com (E.L.-M.); eholgadomartin@gmail.com (E.H.); 4 Department of Pathology, Hospital Ramón y Cajal, 28034 Madrid, Spain; emorenomoreno92@gmail.com; 5 Facultad de Medicina, Universidad de Alcalá de Henares, 28029 Madrid, Spain

**Keywords:** lobular carcinoma, pleomorphic, NGS, *ERBB2*

## Abstract

*Background:* Characterisation of molecular alterations of pleomorphic lobular carcinoma (PLC), an aggressive subtype of invasive lobular carcinoma (ILC), have not been yet completely accomplished. *Methods:* To investigate the molecular alterations of invasive lobular carcinoma with pleomorphic features, a total of 39 tumour samples (in situ and invasive lesions and lymph node metastases) from 27 patients with nuclear grade 3 invasive lobular carcinomas were subjected to morphological, immunohistochemical and massive parallel sequencing analyses. *Results:* Our observations indicated that invasive lobular carcinomas with pleomorphic features were morphologically and molecularly heterogeneous. All cases showed absence or aberrant expression of E-cadherin and abnormal expression of β-catenin and p120. *CDH1* (89%), *PIK3CA* (33%) and *ERRB2* (26%) were the most common mutated genes. *ERBB2* mutations preferentially affected the tyrosine-kinase activity domain, being the most frequent the targetable mutation p.L755S (57%). We also observed higher frequency of mutations in *ARID1B*, *KMT2C*, *MAP3K1*, *TP53* and *ARID1A* in PLC than previously reported in classic ILC. Alterations related to progression from in situ to invasive carcinoma and/or to lymph node metastases included *TP53* mutation, amplification of *PIK3CA* and *CCND1* and loss of ARID1A expression. *Conclusions:* The high frequency of *ERBB2* mutations observed suggests that *ERBB2* mutation testing should be considered in all invasive lobular carcinomas with nuclear grade 3.

## 1. Introduction

Invasive Lobular Carcinoma (ILC) is the second most prevalent histologic subtype of breast cancer, constituting up to 10–15% of all cases [1]. Interestingly, the incidence of ILC has increased significantly compared with that of invasive ductal carcinoma of non-special type (IDC) since the 1980s, probably due to the use of hormone replacement therapy during menopause [2]. There are several variants of ILC with lack of cell-cell cohesion as a common feature: classic ILC, solid ILC, alveolar ILC, tubulo-lobular ILC and pleomorphic ILC (PLC) [2]. PLC is an uncommon but clinically important form of ILC, it was first described as a more aggressive variant of ILC compared with the classic subtype [3,4]. In terms of epidemiology, PLC represents less than 1% of all breast cancer and about 15% of ILC and it is associated with older age and menopausal status. Despite having a growth pattern similar to classic ILC, PLC shows both a higher degree of cellular atypia and pleomorphism and a higher mitotic rate, than classic ILC. Additionally, PLC may show apocrine or histiocytoid differentiation and it may be composed of signet ring cells [2].

The key molecular hallmark of ILC is the loss of the epithelial specific cell-cell adhesion molecule E-cadherin, encoded by *CDH1* [5]. Genetic mutations involving loss of function have been described in all subtypes of ILC [6], ranging between 50–60% of ILC cases. Deleterious mutations in *CDH1* are usually accompanied by the loss of 16q, where the gene is located [7]. Thus, there is a complete loss of E-cadherin, conferring the highly non-cohesive morphological characteristics of this type of tumour [1]. In addition, the loss of E-cadherin expression is also observed in LCIS, so *CDH1* mutations/alterations are expected to be early events in this subtype.

More recently, three large studies based on next generation sequencing (NGS) techniques have reported the molecular characteristics of ILC. These studies have demonstrated that there are molecular differences between ILC and IDC and that the PI3K pathway is the most frequently altered due to mutations in *PIK3CA*, *PTEN* or *AKT1* [8]. Although not specifically stated, most cases included in this series corresponded to classic ILC. Out of these three studies, only Desmedt et al. [9] tried to associate molecular alterations with specific morphological features, such as nuclear pleomorphism, growth pattern or mitotic index.

At present we partially lack molecular information regarding ILC histological subtypes. PLC has been characterised previously using immunohistochemistry, single gene analysis and microarray-based comparative genomic hybridization (aCGH) [9,10,11,12]. These analyses have confirmed the loss of E-cadherin expression and 16q loss of heterocigosity (LOH) [9] in PLC. Additionally, PLC carries molecular alterations typical of high-grade IDC, such as *HER2* and *TP53* alterations, more frequently than classic ILC [10,11,12]. Lastly, two NGS studies have specifically analysed the molecular alterations in PLC in two small series of 8 and 16 tumours respectively [13,14].

PLC is more aggressive than other forms of ILC. In spite of previous studies, molecular characterisation of PLC has not yet been fully accomplished. Thus, we have selected PLC tumours from 27 patients to be characterised by NGS using a specific gene panel to target the most frequently mutated genes and altered chromosomal regions in breast cancer. To gain insight into the molecular alterations involved in tumour progression, we analysed the molecular differences between the in-situ and the invasive components in 7 tumours and between the invasive component and the lymph node metastasis in 4 tumours.

## 2. Materials and Methods

Compliance with Ethical Standards: All procedures performed in studies involving human participants were in accordance with the ethical standards of the institutional and national research committee and with the 1964 Helsinki declaration and its later amendments or comparable ethical standards.

### 2.1. Case Selection

A total of 39 tumour samples from 27 patients diagnosed between 2010 and 2017 were selected from the Pathology Department in Ramón y Cajal Hospital (Madrid, Spain). Cases were included in the study if they had a growth pattern suggestive of ILC, nuclear grade 3, absence or abnormal E-cadherin expression (see Section 3) and tissue available for additional immunohistochemical and molecular studies (Supplementary Table S1). The study included 27 invasive, 7 in-situ, 4 lymph node metastasis and 1 relapse lesions.

Histological evaluation was performed according to WHO recommendations and included tumour size, growth pattern (classic, solid, alveolar or trabecular) and cellular morphology (classic, apocrine or histiocytoid). All cases were graded according to the three–tiered Nottingham histological grading system [2].

### 2.2. Immunohistochemistry

The antibodies used are shown in Supplementary Table S2. Immunostaining was performed using the EnVision detection system (K5007, Dako, Glostrup, Denmark). A cut-off value of 1% was used to define ER, PR or AR positivity. Ki67 index was defined as the percentage of positive cells independently of intensity. HER2 expression was interpreted according to 2018 American Society of Clinical Oncology and the College of American Pathologists (ASCO-CAP) guidelines [15]. E-cadherin, β-catenin and p120 expression was classified as membranous, cytoplasmic or nuclear. Membranous expression was considered to be preserved (intense expression in more than 75% of neoplastic cells), reduced or absent as previously reported [16]. For cyclin D1, ARID1A and ARID1B nuclear proteins, a 5% cut-off value was used to define positivity. The total number of CD8+ T lymphocytes were visually measured in each tumour microarray, counting intratumoural and stromal lymphocytes altogether. Tumours were considered to be PDL1-positive if PDL1 expression was observed in more than 1% of neoplastic cells.

Tumours were classified in different intrinsic subtypes as Luminal (see Section 3), HER2-enriched (HER2-positive and ER- and PgR-negative) and Triple Negative (HER2-, ER- and PgR-negative) according to Curigliano et al. criteria [17]. Luminal tumours were classified according to Maisonneuve et al. [18] criteria as follow: (1) Luminal A–like tumours are ER-positive and HER2-negative with low Ki-67 expression (<14%) or with intermediate Ki-67 expression (14% to 19%) and high PgR levels (≥20%); (2) Luminal B–like (HER2-negative) tumours are ER-positive and HER2-negative with intermediate Ki-67 expression (14% to 19%) and low PgR levels (<20%) or with high Ki-67 expression (≥20%).

### 2.3. Fluorescent In-Situ Hybridisation (FISH)

HER2 equivocal cases (2+) were subjected to fluorescent in situ hybridisation, using the path vision HER-2 DNA Probe Kit (Abbot Laboratories, City, Country). Results were interpreted according to 2018 ASCO-CAP guidelines.

### 2.4. Massive Parallel Sequencing

To obtain DNA mainly from tumour cells, samples from different lesions were obtained by “punching” paraffin blocks in selected areas previously marked on haematoxylin/eosin (H&E) slides. QIAamp DNA FFPE Tissue Kit (Qiagen, Valencia, CA, USA) was used to extract DNA from all samples. Quality of DNA samples was measured using TapeStation (Agilent 2200 TapeStation, Santa Clara, CA, USA), whereas quantification was done by QUBIT 2.0. (Thermo Fisher Scientific Qubit 2.0 Fluorometer, Waltham, MA, USA).

A custom gene panel was designed using SureDesign platform by Agilent Tech. (Santa Clara, CA, USA) to consistently target 34 genes (AKT1, ARID1A, ARID1B, BRCA1, BRCA2, CASP8, CCND1, CDH1, ERBB2, ESR1, FGFR1, GATA3, GRB7, GSDMB, MAP2K4, KRAS, MAP3K1, MLL3, MYC, NCOR1, NF1, PGAP, PIK3CA3, PNMT, PTEN, RB1, SF3B1, STARD3, TBX3, TCAP, TP53, VGLL1, ZNF217, ZNF703) and regions in chromosome 8 (targeting amplification of FGFR1 and MYC), chromosome 11 (targeting amplification of CCND1), chromosome 17 (targeting amplification of ERBB2) and chromosome 20 (targeting amplification of ZNF217). For library construction, a modified protocol for Agilent SureSelect^QXT^ was selected [19], based on the enzymatic fragmentation of DNA and subsequent probe-mediated hybridisation capture [20]. Sequencing of equimolar libraries was performed using the Miseq v2 2 × 150 bp method by Illumina Inc., San Diego, CA, USA.

Bioinformatics analysis was carried out using a specific pipeline using Novoalign (http://www.novocraft.com/products/novoalign/) as aligner and VarScan [21] as variant-caller, with no filters. Variant annotation was performed using the VEP from Ensembl version 88 (http://www.ensembl.org/info/docs/tools/vep/index.html), which corresponds to hg38 version of the human reference genome. Variants were latterly filtered using the functional information (taking only deleterious variants), the variant allele frequency (>0.05) and the strand-bias from both the variant and the reference allele. If normal tissue was available, those variants also present in the normal component were ruled out. Finally, visual inspection was performed as the final selection criterion. In addition, dubious variants were confirmed or ruled out using Sanger sequencing. CONTRA package [22] was used to analyse the genome instability using a baseline obtained from several normal diploid FFPE samples (from normal Fallopian tubes) used as control samples.

### 2.5. Estimation of LOH Status in CDH1

Since our gene panel was not designed to identify losses on the *CDH1* chromosomal region, we aimed to estimate the LOH status of *CDH1* for each sample. Although two different polymorphisms (rs3743674 and rs1801552) were constitutively identified in our tumour set, they failed to estimate the LOH status in the region, due to the allele frequencies in the population and the apparently linked status of their alleles.

Therefore, we developed a simple formula based on the percentage of tumour cells:

$$Ti\;=\;\frac{\%tumour_{cells}}{2\;-\;\%tumour_{cells}}$$

This *Ti* (Tumoural index) was then compared to the frequency of the mutated allele in our NGS data, giving an approximation of the LOH status of *CDH1*. Considering that the percentage of tumour cells could be overestimated and that the frequency of the variant allele observed by NGS data could be underestimated, we considered a −0.17 × Ti deviation as acceptable. See Supplementary File 1 for complete mathematical explanations.

## 3. Results

### 3.1. Clinicopathological Features

Clinicopathological and immunohistochemical features of all samples are shown in Supplementary Table S1 and summarised in Table 1. The average age at diagnosis was 63 y/o (range 39 to 87), being 63% of patients older than 60 years.

**Figure 1:**
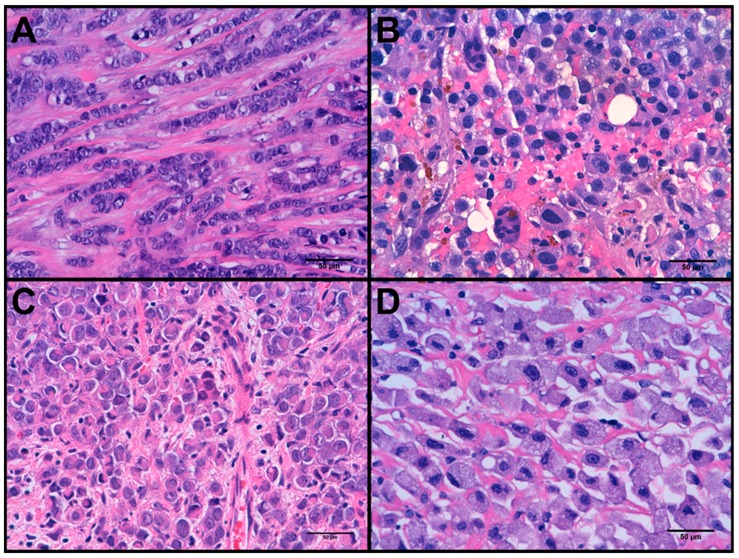
Histological variants of pleomorphic lobular carcinomas (PLC). (**A**) PLC with no other cell type. (**B**) PLC with osteoclast-like giant cells. (**C**) Apocrine PLC. (**D**) Histiocytoid PLC.

All 27 PLCs had high nuclear grade (G3) and no tubular formation, hence all were classified as histologic grades 2 (63%) or 3 (37%), depending on their mitotic count. The predominant pattern of growth was trabecular (48%), followed by solid (37%) and classic (15%). Regarding cytomorphology (see Figure 1), 7 cases (26%) showed histiocytoid differentiation and 3 cases (11%) apocrine differentiation. This series included an uncommon case rich in osteoclast-like cells [23]. According to classification criteria [17,18], 17 PLCs (~63%) were luminal A, 5 (~19%) PLCs were luminal B, 4 (~15%) PLCs were triple negative and 1 (~4%) PLC was HER2-enriched.

**Figure 2:**
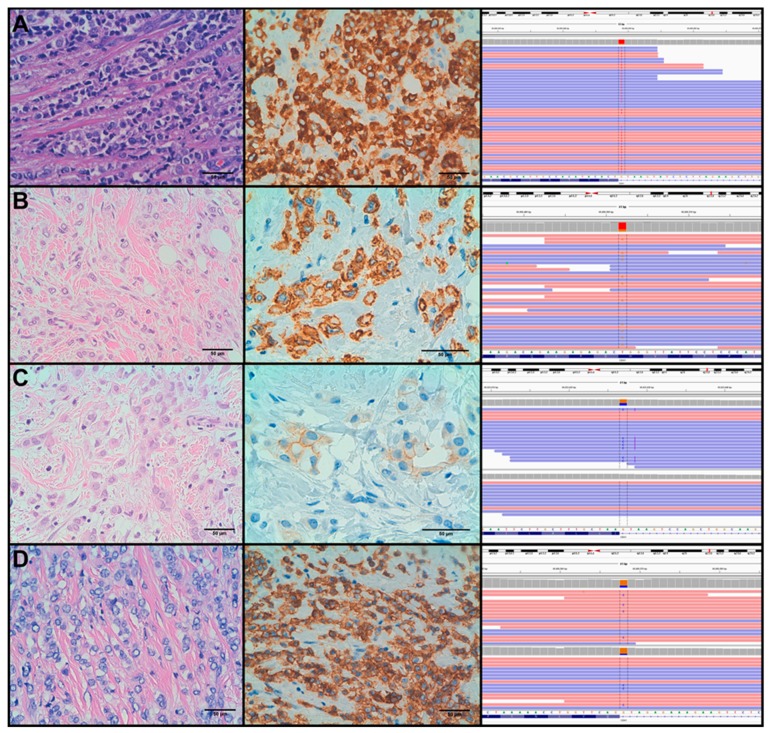
PLC cases showing aberrant expression of E-cadherin: PLC-5 (**A**), PLC-7 (**B**), PLC-18 (**C**) and PLC-22 (**D**). From left to right: H&E, E-cadherin staining and IGV view of *CDH1* mutations. In PLC-18 and PLC-22, reads and bases are shown for invasive (top) and in-situ (bottom) components.

As to E-cadherin expression, 23 PLCs showed complete absence of expression. The remaining four cases showed different patterns of abnormal expression (see Figure 2): three cases had aberrant cytoplasmic expression and one PLC showed severe reduction of membranous expression.

Consistent with the abnormal E-cadherin expression patterns described, all cases showed abnormal β-catenin and p120 expression patterns. Regarding β-catenin, 63% of PLCs showed complete absence of expression and the remaining cases had abnormal cytoplasmic expression. p120 cytoplasmic expression was observed in 92% of the cases and of note, 22% of PLCs displayed both cytoplasmic accumulation and nuclear expression.

Regarding other markers, no cases showed p53 overexpression or a null pattern of expression. All tumours expressed ARID1B and CK19. 70% of PLCs expressed androgen receptor and 26 out of 27 (92%) PLCs expressed ARID1A and cyclin D1. PDL1 was positive in only six PLCs, considering 1% as the cut-off. The average number of CD8+ lymphocytes per tumour was 35 (range 7–90).

### 3.2. Mutation Analysis

**Figure 3:**
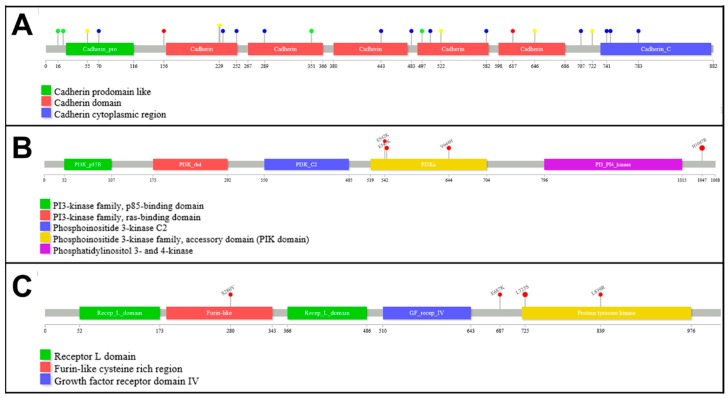
Distribution of somatic mutations along (**A**) *CDH1*, (**B**) *PIK3CA* and (**C**) *ERRB2* genes. Red lollipops represent missense mutations while green lollipops represent nonsense mutations, blue lollipops represent frameshift mutations and yellow lollipops represent splice-affecting mutations.

Variant analysis results are shown in Supplementary Table S3. A total of 78 somatic variants and 8 amplifications were identified among the 27 invasive samples. As expected, most samples (24 out of 27, 88.89%) had mutations in *CDH1* (see Table 2), the most frequently mutated gene in this series. The distribution of mutations across *CDH1* is shown in Figure 3A. Interestingly, 12 out of the 25 (48%) mutations were indels, 6 (24%) mutations were located in the conserved donor site of splice regions, 4 (16%) were nonsense mutations and 2 (8%) were missense mutations. According to the wTi we calculated for 24 cases in which the percentage of tumour cells could be estimated (see Supplementary Table S4), we considered that up to 18 out of 21 (85%) cases with mutations in *CDH1* could probably also carry LOH in the region.

Together with *CDH1*, *PIK3CA* and *ERRB2* were the most common mutated genes in this series and showed mutations in 9 (33%) and 7 (26%) tumours respectively. In addition, both *PIK3CA* and *ERRB2* were amplified in one case respectively. The mutation distribution across *PIK3CA* and *ERBB2* are shown in Figure 3B,C respectively. Most mutations in *PIK3CA* were found in hotspot positions: p.H1047R (58%), located in the phosphoinositide-3-kinase domain; and p.E545K (17%) and p.E542K (8.5%), both located in the protein kinase-C homology domain. We also found the same mutation (p.Y644H) in 2 different cases, located in the helical domain, which are probably germline variants. Similarly, we found a predominant hotspot mutation in *ERBB2* (p.L755S, 57%) and other 2 mutations (p.E717K and p.L869R) also affecting the tyrosine-kinase activity domain. We also found a mutation (p.S310Y) located in the binding-site domain.

Mutations in other genes involved in the PI3K/AKT pathway included mutations in *MAP3K1* (present in 5 cases, 19%) and *AKT1* (present in 2 cases, 8%). Mutations in *PIK3CA*, *ERBB2* and *AKT1* (present in a total of 16 cases) tend to be mutually exclusive (only cases PLC9 and PLC19 carried mutations in both *PIK3CA* and *ERBB2*). In contrast, all *MAP3K1* mutations occurred in cases with mutations in *PIK3CA* or *ERBB2*.

Mutations in *TP53* occurred in 5 cases (18.52%). Interestingly, whereas 2 cases had mutations producing truncated proteins (c.550_551delGA and p.R342*), the remaining 3 cases displayed missense mutations (p.R175H, p.Y220C and p.E286K) affecting the DNA binding domain.

Mutations in the chromatin remodelling genes *ARID1B* and *ARID1A* were found in 6 (22%) and 4 (15%) tumours, respectively. Mutations in both genes were mutually exclusive except in one case (PLC15). Mutations in the gene encoding the histone H3 lysine 4 methyltransferase *KMT2C* were found in 5 cases (19%).

As for amplifications, in addition to the previously mentioned cases with *ERBB2* and *PIK3CA* amplification, we observed that the *CCND1* region was amplified in 3 cases (11%) and *FGFR1* in 2 cases (7%).

In addition to somatic mutations, we observed a set of infrequent germline variants (Supplementary Table S5) that were overrepresented in our series. The extreme overrepresentation of *BRCA2* variants and *ERRB2* variant rs141116145 (p.A356D) is noteworthy, since two different cases present the variant allele while the expected number of carriers in our set was 0.05 according to Hardy-Weinberg equilibrium (see Supplementary Table S5). Interestingly, PLC13 carried the pathogenic mutation c.3860delA in *BRCA2* (confirmed as a germline mutation) and had a family record in which her mother had an ovary carcinoma and her daughter debuted with breast carcinoma at the age of 30.

### 3.3. Molecular Alterations and Histopathological/Immunohistochemical Features

Regarding the relationship between molecular alterations and IHC features, it is interesting to note that aberrant expression patterns of E-cadherin can be found in *CDH1*-mutated tumours that can be mistaken with positive E-cadherin staining (see Figure 2). We observed an aberrant expression pattern of E-cadherin in 4 cases: PLC5, PLC7, PLC18 and PLC22 (see Supplementary Table S1 and Figure 2). In 3 out of the 4 cases we identified mutations in *CDH1* that affected canonical splicing sites (c.531+1G > C, c.687+1G > T and c.2164+1G > C), according to in-silico assays (data not shown). The remaining case portrayed a missense mutation (p.W156G) which functional effect on the protein has not been elucidated. On the other hand, we found other three cases (PLC10, PLC16 and PLC27) portraying mutations that affected canonical splicing sites (c.1565+1G > A, c.163+2T > G and c.1936+1G > C), which presented negative expression of E-cadherin.

**Figure 4:**
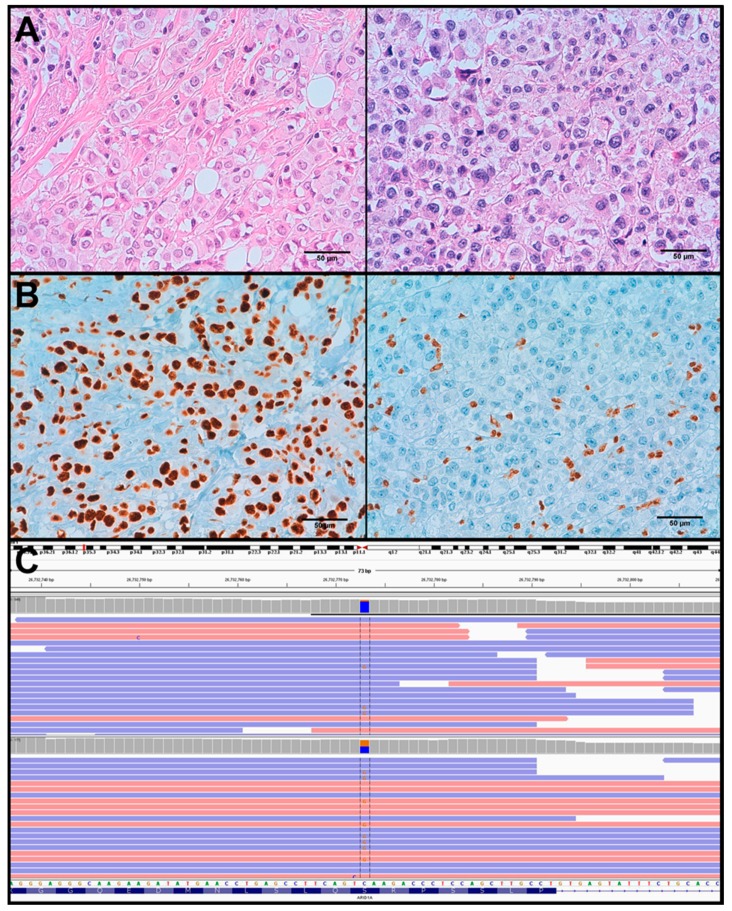
PLC24 sample. (**A**) H&E staining of invasive (left) and metastatic (right) components. (**B**) ARID1A positive staining of invasive component (left) and ARID1A negative staining of metastatic component (right). (**C**) IGV view of *ARID1A* S634* mutation frequency (orange) found by NGS sequencing in invasive (top, depth ~500) and metastatic (bottom, depth ~170) components.

In spite of a high frequency of *ARID1B* and *ARID1A* mutations in this series, only one tumour showed absence of expression of ARID1A. Similarly to *CDH1*, LOH status of the *ARID1A* region was estimated in the four cases presenting mutations in this gene and we concluded that LOH could probably occur in only two cases (see Supplementary Table S4). Interestingly, lack of ARID1A expression was confirmed only in the metastatic component of PLC24 (see Figure 4), validating the LOH status of the *ARID1A* region in this sample and confirming the pathogenic role of the nonsense p.S634* mutation.

It is important to highlight that mutations in *ERBB2* and *TP53* did not produced abnormal patterns of IHC features.

### 3.4. Molecular Alterations Related to Tumour Progression and Relapse

For nine patients, we obtained tumour samples from different localizations: the in-situ and the invasive components in four patients; the invasive breast tumour and a lymph node metastasis in three patients; the in-situ and invasive breast components and a lymph node metastasis in one patient and the in-situ primary tumour and the invasive relapse lesion occurring almost five years later in one patient. Comparative results of variants identified in these cases are shown in Supplementary Table S6. Molecular similarities among components highlighted *TP53* mutations and amplifications of *PIK3CA* and *CCND1* as progression hallmarks. It is interesting to note that we were able to detect *ERBB2* mutations in the in-situ component of two cases of PLC that carried this alteration in the invasive component too. Nine (33%) out of 27 patients relapsed after a mean follow-up of 44 months; and one (4%) patient died after 19 months of follow-up. We did not find any pathological feature or molecular alteration associated with tumour relapse.

## 4. Discussion

In the current study, the morphological, immunohistochemical and molecular characterisation of a set of invasive lobular carcinomas of the breast with pleomorphic features (at least extensive nuclear grade 3) have all been performed. This study indicates that invasive lobular carcinomas with grade 3 nuclei are heterogeneous, both at the morphological and molecular levels. In fact, we observed different morphological variants, such as histiocytoid, apocrine and osteoclast-rich carcinomas. All intrinsic subtypes of breast cancer were represented in this series, although most tumours were classified as luminal A with grade 2, probably due to our selection criteria (high nuclear grade and loss or aberrant expression of E-cadherine, independently of the mitotic rate).

Regarding mutational analysis, our study showed some differences with respect to previous NGS studies in ILC. Thus, although *CDH1* was the most frequently mutated gene in different series of ILC, the frequency of tumours carrying mutations in *CDH1* was about 88% in our series whereas frequencies varied from 43% to 65% in the other studies [1,8,24]. These differences are most likely related to case selection rather than to intrinsic biological features of PLC. Thus, all our cases were selected not only according to morphological features but also for the lack or aberrant E-cadherin expression. In this sense, only one of the three previous NGS series analysing ILC did evaluated the immunohistochemical expression of E-cadherin in a limited number of cases, suggesting that possibly not all cases included in previous studies represented *bona fide* examples of ILC. In addition, even though all PLC in Zhu et al.’ series had negative E-cadherin staining, the percentage of cases that carried *CDH1* mutations was 59% [14].

In our series, we did not detect E-cadherin mutation in 3 out of 27 cases with absence of E-cadherin expression. Previous studies suggested that in these cases the loss of E-cadherin expression could be due to a combination of *CDH1* LOH and promoter hypermethylation [16]. However, Ciriello et al. has recently questioned this hypothesis, suggesting that promoter hypermethylation is not a feature of ILC [1]. In our series, although we did not studied *CDH1* promoter hypermethylation, we estimated the presence of LOH in 16q22 in 85% of the tumours, which is in agreement with previous studies [25].

One interesting finding in our study is the observation of 4 cases (15%) with morphological characteristic of PLC, an abnormal pattern of E-cadherin expression and the presence of *CDH1* mutations. It has been previously reported that 16% of ILC retained some E-cadherin expression and that, in these cases, abnormal expression of catenins, such as β-catenin or p120, was observed [26]. Our study suggested that certain types of *CDH1* mutations, especially splice site mutations located in specific domains, are more prone to be related to aberrant E-cadherin expression.

An important finding in our study, with potential therapeutic implications, is the high frequency (26%) of *ERBB2* mutations observed in PLC. Previous NGS studies reported a frequency of *ERBB2* mutations in about 5% of unselected ILC [1,8,24]. However, Desmedt et al. reported a correlation between *ERBB2* mutations and histological grade, in accordance with our results. In addition, in a study using conventional Sanger sequencing, Lien at al. [10] observed a frequency of 20.8% of *ERBB2* mutations in PLC compared to only a 2% in classic ILC. Finally, Zhu et al. [14] in their recent NGS study of 16 ILC observed that *ERBB2* was mutated in 17% of the samples. Our results confirmed the important role of *ERBB2* gain-of-function mutations in high-grade invasive lobular breast carcinoma, in agreement with previous observations [27]. Of note, *ERBB2* activating mutations are considered targetable by anti-ERBB2 drugs [28] and other alternatives could be beneficial for these patients [29]. A comparative study of the frequency of activating mutations in *ERBB2* is shown in Table 3. In agreement with previous studies, we did not observe that *ERBB2* mutations were associated with a higher risk of relapse [27].

Alterations in the phosphatidylinositol 3-kinase (PI3K) pathway were present in 13 cases (50%), with mutations in *PIK3CA* (9 cases, one of them with a combination of gene mutation and amplification), *MAP3K1* (5 cases) and *AKT1* (2 cases). As expected, mutations in *PIK3CA* and *AKT1* were mutually exclusive [30] but 3 *MP3K1* mutations were associated with *PIK3CA* mutations. Whereas the frequency of *PIK3CA* mutations in the current series is similar to those reported in other ILC NGS studies, the frequency of *MAP3K1* mutations seems to be higher (19% vs. 5–6%) [1,8,24]. This finding is concordant with the study by Zhu et al. reporting that *MAP3K1* mutations occurred at a higher frequency in PLC than in ILC [14].

Whereas a unique mutation was found in *AKT1* (p.E17K), different known activating mutations were present in *PIK3CA* (p.E545K, p.E542K and p.H1047R). Moreover, another different case (PLC-8) showed the two most frequent mutations in *PIK3CA*, p.E545K and p.H1047R, a phenomenon that seems to be infrequent. Interestingly, the frequency of the variant alleles in the tumour were ~11% for p.E545K and ~26% for p.H1047R, denoting that the former could have been acquired at a later stage in a different chromosome in a specific clone, suggesting clonal diversity.

Additionally, several transcriptional regulators were recurrently mutated in the current series, such as *ARID1A*, *ARID1B* and *KMT2C*. Regarding *ARID1A* and *ARID1B*, although we observed a high frequency of mutations, the immunohistochemical analysis showed a normal expression pattern in most cases. The discordance between the genomic and the immunohistochemical study can be due to different reasons, such as lack of a second hit (i.e., LOH) in most cases, retaining of a non-functional protein that is recognized by the antibodies used, or passenger mutations with no or little impact in gene function. Only one sample with an *ARID1A* mutation and LOH showed ARID1A loss of expression. Interestingly, loss of ARID1A expression was observed in the lymph node metastasis but not in the primary tumour, suggesting a role of this alteration in tumour progression.

In the TCGA study on breast cancer [31], *KTMC2*/*MLL3* was found to be mutated in the 7% of cases, with little differences amongst subtypes (5–8%). These percentages are similar to those reported in other ILC studies [1,8,24]. However, the percentage of cases in which we identified mutation in *KMT2C* was almost 20%, which can contribute to the aggressiveness and dedifferentiation of PLCs due to stemness properties acquired through the loss of *KMT2C* activity [32]. In the study by Zhu et al. [14], *KTMC2* was mutated in 35% of PLC and this percentage was significantly higher than previously observed in ILC.

In addition to the molecular characterisation of PLCs, we also aimed to check the molecular similarities between different lesions from the same case with the purpose of finding out possible progression hallmarks. As expected, the similarity between in-situ and invasive components of the same case was high, although some differences were shown (see Supplementary Table S6). We observed that mutations in *TP53* and amplification of 11q13 region were differential features between invasive and in-situ components, highlighting the role of these genes in aggressive tumours as previously stated [33,34].

Germline mutations in *BRCA1* and *TP53* have been reported to be predominantly associated with invasive ductal carcinomas, while *BRCA2* mutations have been related to both ductal and lobular cancers [35]. In addition, a previous study has reported an excess of PLC among *BRCA2*-mutation carriers [36]. We identified one case out of 27 (~4%) portraying a deleterious germline mutation in *BRCA2*, with familial history of breast and ovarian cancer. Moreover, we identified a set of infrequent germline mutations with an in-vitro deleteriousness role that are normally excluded from molecular characterisation studies (see Supplementary Table S5). We observed that several variants were not expected to appear in such a small set of cases. Among them, some variants that are considered of “uncertain significance” in familial breast cancer studies testing *BRCA1* and *BRCA2*. Other germline variants were located in *ESR1*, *ERBB2*, *PIK3CA*, *KMT2C* and *ZNF217*, genes considered of great importance in the development and outcome of breast cancer [1,8,31,37,38,39,40,41]. These infrequent germline variants could be risk factors for the development of PLC, although further studies are necessary to confirm their role in breast cancer development.

## 5. Conclusions

PLC shows a high degree of heterogeneity from a morphological and molecular point of view. In addition to a high percentage of *CDH1* mutations, some of which were associated with an aberrant E-cadherin expression pattern, these tumours seem to be characterized by higher frequency of mutations in genes related to aggressive behaviour, such as *ERBB2*, *TP53* and *KMT2C*. The high frequency of treatable *ERBB2* mutations in this and other previous series suggest that *ERBB2* mutation testing should be considered in all ILC with nuclear grade 3.

## Figures and Tables

**Table 1:** Clinicopathological features of the 27 patients.

Age at Diagnosis (Years)	#Cases	Receptor Status	#Cases
<35	0	ER+	20
35–49	4	ER−	7
50–69	13		
70+	10	PgR+	16
**Tumour size**		PgR−	11
N/A	2		
<1 cm	1	AR+	22
1–2 cm	7	AR−	5
2–5 cm	15	**Ki67**	
>5 cm	2	<14	20
**Lymph node status**		14–30	6
N0	11	30+	1
N1	11	**HER2**	
N3	4	0/+/++	26
NX	1	+++	1
**Tumour grade**		**E-cadherin status**	
G2	17	-	23
G3	10	aberrant	4
**Subtype**			
Luminal A	17		
Luminal B	5		
Triple Negative	4		
HER2 enriched	1		

**Table 2:** Comparative results with published data for gene mutation frequency.

Gene	Number of Mutations Found in Current Study	%Cases Mutated				
		Current Study (PLC, *n* = 27)	Zhu et al. [14] (PLC, *n* = 17)	Desmedt et al. [8] (ILC, *n* = 413)	Michaut et al. [24] (ILC, *n* = 144)	Ciriello et al. [1] (ILC, *n* = 127)
**CDH1**	25	89%	59%	65%	43%	65%
**PIK3CA**	12	33%	53%	43%	35%	48%
**ERBB2**	7	26%	18%	5%	4%	4%
**ARID1B**	6	22%	-	0%	5%	6%
**KMT2C**	5	19%	35%	8%	10%	7%
**MAP3K1**	5	19%	35%	5%	5%	6%
**TP53**	5	19%	12%	7%	4%	8%
**ARID1A**	4	15%	6%	6%	7%	17%
**CCND1_amp**	3	11%	12%	38%	15%	17%
**AKT1**	2	7%	6%	4%	5%	2%
**FGFR1_amp**	2	7%	-	25%	8%	9%
**GATA3**	2	7%	6%	7%	5%	5%
**NF1**	2	7%	23%	0%	4%	4%
**TBX3**	2	7%	23%	13%	8%	9%
**ERBB2_amp**	1	4%	6%	0%	4%	7%
**PIK3CA_amp**	1	4%	-	0%	1%	2%
**BRCA2**	1	4%	0%	2%	4%	-
**CASP8**	1	4%	-	0%	1%	1%
**NCOR1**	1	4%	23%	0%	7%	6%
**PGAP3**	1	4%	12%	0%	0%	-
**RB1**	1	4%	0%	0%	3%	6%

**Table 3:** *ERBB2* mutations in invasive lobular carcinoma.

Gene	Mutation	Present Study		Available Studies		
		Cases	Frequency	Cases	Frequency	References
ERBB2	p.L755S	4	57.1%	24	33.3%	[1,8,24,27]
	p.L869R	1	14.3%	4	5.6%	[8]
	p.S310Y	1	14.3%	2	2.8%	[27]
	p.E717K	1	14.3%	-	-	-
	p.V777L			10	13.9%	[1,8,14,24]
	p.D769Y			5	6.9%	[8,24]
	p.S310F			4	5.6%	[8,11,24]
	p.A775_G776insYVMA			3	4.2%	[24]
	p.L755_T759del			3	4.2%	[10,24]
	p.I767M			2	2.8%	[24,27]
	p.R678Q			2	2.8%	[1,24]
	c.1647-2A > G			1	1.4%	[24]
	c.2923_2923delG			1	1.4%	[24]
	p.A771V			1	1.4%	[10]
	p.A775V			1	1.4%	[10]
	p.E1021K			1	1.4%	[8]
	p.L755M			1	1.4%	[1]
	p.L755W			1	1.4%	[1]
	p.R434Q			1	1.4%	[14]
	p.R978C			1	1.4%	[8]
	p.S305C			1	1.4%	[1]
	p.T791I			1	1.4%	[10]
	p.V697L			1	1.4%	[8]
	p.V842I			1	1.4%	[24]

## Supplementary Materials

The following are available online at http://www.mdpi.com/2072-6694/11/1/74/s1, Supplementary file 1: Complete mathematical explanations. Table S1: Clinicopathological and immunohistochemical features of the 27 PLC studied, Table S2: List of antibodies used to characterise PLC tumours, Table S3: List of mutations found in the invasive component of the 27 PLCs subjected to NGS analysis, Table S4: Estimation of LOH status based on the calculation of Ti, Table S5: List of infrequent germline variants identified in the 27 PLCs subjected to NGS analysis, Table S6: List of mutations found in the different components of 10 PLCs subjected to NGS analysis.
