# Supplementary material for: High Frequency of ERBB2 Activating Mutations in Invasive Lobular Breast Carcinoma with Pleomorphic Features
Source: Cancers (Basel). 2019 Jan 11;11(1):74. doi: 10.3390/cancers11010074 (PMC6356653; doi:10.3390/cancers11010074)
Supplement: Supplementary file 1 [file cancers-11-00074-s001.zip › cancers-399636 -SI.pdf]

# Supplementary Materials: High Frequency of *ERBB2* Activating Mutations in Invasive Lobular Breast Carcinoma with Pleomorphic Features

**Juan Manuel Rosa-Rosa, Tamara Caniego-Casas, Susanna Leskela, Eva Cristobal, Silvia González-Martínez, Esther Moreno-Moreno Elena López-Miranda, Esther Holgado, Belén Pérez-Mies, Pilar Garrido and José Palacios**

**DEVELOPMENT OF Ti:**

Ti = %tumour_cells/(2 − %tumour_cells)

Let's assume a tumour with both tumoural and normal (wild type cells):

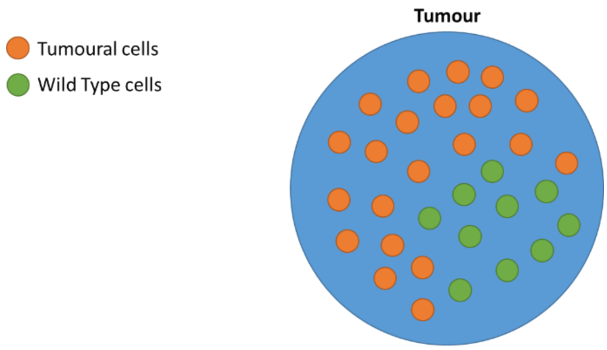


If tumoural cells carry the deletion of a chromosomal region and a mutation in a gene located in that chromosomal region, then we can say that the total number of chromosomes (#chr) is equal to the sum of the number of wild type chromosomes (#chr_wt) plus the number of mutated chromosomes (#chr_m):

#chr = #chr_wt + #chr_m;

since normal cells (normal_cells) carry 2 copies of the chromosomal region and tumoural cells (tumour_cells) carry 1 copy of the chromosomal regions, we can say that:

#chr_wt = 2 × #normal_cells;

#chr_m = 1 × #tumour_cells;

we do not know the total number of cells (#cells) in our tumour, but we know the percentage of normal (%normal_cells ) and tumoural cells (%tumour_cells), thus we can say that:

1 = %normal_cells + %tumour_cells;

%normal_cells = 1 − %tumour_cells;

#normal_cells = %normal_cells × #cells = (1 − %tumour_cells) × #cells;

#tumour_cells = %tumour_cells × #cells;

#chr_wt = 2 × (1 − %tumour_cells) × #cells;

#chr_m = 1 × %tumour_cells × #cells

then #chr can be calculated as:

#chr = #chr_wt + #chr_tm;

#chr = 2 × (1 − %tumour_cells) x #cells + %tumour_cells × #cells = (2 – 2 × %tumour_cells + %tumour_cells) × #cells;

$$\#chr = (2 - \%tumour\_cells) \times \#cells$$

and then we can calculate the ratio of mutated chromosomes or tumoural index (Ti) as follows:

$$Ti = \#chr\_m / \#chr:$$

$$Ti = (\%tumour\_cells \times \#cells) / ((2 - \%tumour\_cells) \times \#cells);$$

$$Ti = \%tumour\_cells / (2 - \%tumour\_cells)$$

For example, if the percentage of tumour cells in a tissue is estimated in 60%, then the expected frequency of a somatic variant (Vi) would be:

$$V_i = 0.6 / (2 - 0.6) = 0.6/1.4 \approx 0.43$$

then if the observed frequency of a somatic variant is similar to 0.43 then it may indicate that this somatic variant is probable homozygous due to LOH in the chromosomal region. Thus the range of expected frequencies for homozygous somatic mutations in LOH regions would be:

| %tumour_cells | Vi |
|---|---|
| 0.2 | 0.11 |
| 0.3 | 0.18 |
| 0.4 | 0.25 |
| 0.5 | 0.33 |
| 0.6 | 0.43 |
| 0.7 | 0.54 |
| 0.8 | 0.67 |
| 0.9 | 0.82 |

Since %tumour_cells can be overestimated and the real frequency of a somatic mutation (V) can be underestimated, a probable deviation for Vi is expected. Based on validation results (data not shown), we estimated a theoretical deviation of 0.17 for Vi, thus we weighted Vi by 0.17, obtaining wTi for the 23 cases in which %tumour_cells was estimated.

**Supplementary Tables (S1–S6) are in a separate excel file**

© 2019 by the authors. Licensee MDPI, Basel, Switzerland. This article is an open access article distributed under the terms and conditions of the Creative Commons Attribution (CC BY) license (http://creativecommons.org/licenses/by/4.0/).
